# Host-parasite interaction: changes in human placental gene expression induced by *Trypanosoma cruzi*

**DOI:** 10.1186/s13071-018-2988-0

**Published:** 2018-08-24

**Authors:** Christian Castillo, Ileana Carrillo, Gabriela Libisch, Natalia Juiz, Alejandro Schijman, Carlos Robello, Ulrike Kemmerling

**Affiliations:** 1 Programa de Anatomía y Biología del Desarrollo, Instituto de Ciencias Biomédicas, Facultad de Medicina, Universidad de Chile, Santiago, Chile; 2 Molecular Biology Unit, Pasteur Institute and Departamento de Bioquímica, Facultad de Medicina, Universidad de la República, Montevideo, Uruguay; 3 Instituto de Investigaciones en Ingeniería Genética y Biología Molecular “Dr. Héctor Torres”, Buenos Aires, Argentina

**Keywords:** *Trypanosoma cruzi*, Placenta, Global gene expression, Microarray

## Abstract

**Background:**

Chagas disease is caused by *Trypanosoma cruzi*, a parasite endemic to Latin America. Most infections occur in children by vector or congenital transmission. *Trypanosoma cruzi* establishes a complexity of specific molecular parasite-host cell interactions to invade the host. However, most studies have been mainly focused on the interaction between the parasite and different cell types, but not on the infection and invasion on a tissue level. During congenital transmission, *T. cruzi* must cross the placental barrier, composed of epithelial and connective tissues, in order to infect the developing fetus. Here we aimed to study the global changes of transcriptome in the placental tissue after a *T. cruzi* challenge*.*

**Results:**

Strong changes in gene expression profiling were found in the different experimental conditions, involving the reprogramming of gene expression in genes involved in the innate immune response.

**Conclusions:**

*Trypanosoma cruzi* induces strong changes in genes involved in a wide range of pathways, especially those involved in immune response against infections.

**Electronic supplementary material:**

The online version of this article (10.1186/s13071-018-2988-0) contains supplementary material, which is available to authorized users.

## Background

Chagas disease is a zoonotic disease caused by *Trypanosoma cruzi*, a parasite endemic to Latin America. Most infections occur in children by vector or congenital transmission. The prevalence of Chagas disease in pregnant women in Latin America ranges between 5–40% depending on the geographical area and the rate of congenital transmission is estimated to be 1–12% [1]. In addition, due to population mobility, Chagas disease has been increasingly detected in other non-endemic countries and continents (where the vector does not exist) such as the USA, Canada, Australia, Europe and Asia [2, 3]. Congenital transmission, in spite of its low transmission rates, is partially responsible for the progressive globalization of the disease [2, 4, 5]. Importantly, congenital infection is responsible for an estimated 22% of new infections in 2010, making this form of transmission epidemiologically relevant [6].

The parasite presents a complex life-cycle that occurs in both vertebrate and invertebrate hosts, where three major developmental stages are observed: epimastigotes, trypomastigotes and amastigotes. Trypomastigotes constitute the extracellular infective form in mammals where they are able to infect a wide range of nucleated mammalian cells [7]. Interestingly, *T. cruzi* has co-evolved with mammals to establish a complexity of specific molecular parasite-host cell interactions to invade host cells and tissues, to evade the host immune system and to undergo intracellular replication [8]. Key steps in parasite infection include its host cell penetration and replication of the protozoa in the cytoplasm of infected cells. The application of oligonucleotide and cDNA microarray technologies in the study of host-parasite interactions have permitted rapid and unbiased examination of changes in expression of a large number of genes at the level of transcription [9, 10]. However, these studies have been mainly focused on the interaction between the parasite and different cell types, but not on the infection and invasion on a tissue level.

During congenital transmission, *T. cruzi* must cross the placental barrier in order to infect the developing fetus [3, 11]. This anatomical barrier is formed by the trophoblast, a two-layer epithelium which is in direct contact with maternal blood, the fetal connective tissue (villous stroma), the endothelium of fetal vessels and the basal laminae that support the epithelia [3, 12]. Interestingly, the congenital transmission rate for *T. cruzi* is low [4, 13] and it has been proposed that the placenta might play an important role avoiding parasite infection [3].

The study of gene expression profiles during infection constitutes a very powerful tool to analyze global responses of several kinds of cells and tissues, allowing the identification of new genes and/or pathways implicated in the establishment of the infection and pathogenesis as well as possible local tissue responses [3, 10]. Therefore, here we aimed to study the global changes of transcriptome in the placental tissue after *T. cruzi* challenge*.*

## Methods

### Parasite harvesting

Trypomastigotes from *T. cruzi* (Y Strain, *T. cruzi* II) were obtained from previously infected Vero cells (ATCC® CCL-81) grown in RPMI medium supplemented with 5% fetal bovine serum (FBS) and antibiotics (penicillin-streptomycin) at 37 °C in a humid atmosphere at 5% CO_2._ Parasites invaded the cells and replicated intracellularly as amastigotes, after 48–72 h; amastigotes transformed back to trypomastigotes and lysed host cells. The infective trypomastigotes were separated from cellular debris by low speed centrifugation (500× *g*). From the supernatant, the parasites were isolated by centrifugation at 3500× *g*, suspended in RPMI media (without FBS, 1% antibiotics; RPMI 1640, Biological Industries Ltd., Kibbutz Beit Haemek, Israel) and quantified in a Neubauer Chamber [14, 15].

### HPE infection

HPE were obtained from healthy mothers with uncomplicated pregnancies by cesarean delivery. Placentas were processed in a class II laminar flow hood immediately after delivery. The maternal and fetal surfaces were discarded and villous tissue was obtained from the central part of the cotyledons. The dissected explants were washed with sterile PBS in order to get rid of the blood and co-cultivated with *T. cruzi* trypomastigotes in serum free RPMI media. HPE were challenged with 10^5^ or 10^6^ parasites/ml, since these concentrations have been proposed to correlate with low or high parasitaemia, respectively [16]. For validation experiments, LPS (10 ng/ml) was used as positive control. After 2 or 24 h of infection (in order to study early and late placental responses [16–18], explants were collected in RNA later solution (Thermo Fisher Scientific, Waltham, Massachusetts, USA), stored at 4 °C for 24 h and at -80 °C for posterior RNA isolation [19].

### RNA purification and microarray experiment

Total RNA was isolated with a Purelink RNA isolation kit (Thermo Fisher Scientific) according to the manufacturer’s instructions. RNA integrity was analyzed with a Bioanalyzer 2100 (Agilent Technologies, Santa Clara, California, USA) obtaining RNA integrity numbers (RIN) above 8 for all samples (on a scale based on an rRNA *28S*/*18S* ratio where a RIN of 1 corresponds to a totally degraded RNA and 10 to a totally non-degraded RNA). RNA concentration was quantified by spectrophotometry (Nanodrop, Thermo Fisher Scientific). One hundred nanograms of total RNA was reverse-transcribed into cDNA, then transcribed to cRNA and Cy3-labeled with a Low Input Quick Amp-One Color Labeling Kit (Agilent Technologies). The labeled cRNA was purified with an illustra RNAspin Mini Isolation Kit (GE Healthcare, Little Chalfont, UK) and the total yield was measured with a Qubit RNA HS Kit (Thermo Fisher Scientific). Hybridization, washing, assembling of the chips, and scanning were performed according to the manufacturers’ instructions. Briefly, labeled samples were hybridized with SurePrint G3 Human GE 8x60K chips for 17 h at 60 °C in an Agilent hybridization oven at 10× *rpm*. Posterior washing, stabilization and drying procedures were performed according to Agilent’s Low Input Quick Amp Labeling Kit instructions [10].

### Data analysis

Chips were scanned with an Agilent microarray scanner G2565BA; the software Agilent Feature Extraction (version 9.5.1), was used for quality control, data filtering and data normalization. Extracted data from the SurePrintG3 8x60K chips were analyzed using GeneSpring GX 13.0 software. Genes showing a 2-fold change in their expression (or more) with *P* ≤ 0.05 were considered differentially expressed using ANOVA and Benjamini-Hochberg false discovery rate correction for multiple testing. Analysis of interaction networks between upregulated genes in each experimental group was performed with Cytoscape network visualization and integration software and GeneMania open-source gene function prediction service plug-in (http://www.genemania.org/) and visualized by the corresponding Cytoscape software version 3.0.2 plug-ins [20] The weighting of the network attributes was set to Gene Ontology (GO)-based weighting for biological processes. Gene set enrichment analysis (GSEA) was performed with GSEA 3.0 software (Broad Institute, Cambridge, Massachussets, USA) [21]. Each gene set permutation was performed 1000 times, analyses were based on the GO pathways database (http://geneontology.org/) [22] and a normalized enrichment score (NES) was obtained for each gene set. An enrichment map from data obtained with GSEA was generated with the Enrichment Map plug-in for Cytoscape 3.0 [23]. NES and false discovery rate (FDR) q-value were considered as parameters for the analysis. The NES value allows the comparison of analysis results across gene sets because it considers differences in gene sets sizes corrected by the size of the expression dataset; the FDR represents the estimated probability that a gene set with a given NES represents a false positive finding [22].

### RT-qPCR

One hundred picograms of total RNA was retro-transcribed to cDNA using an M-MLV Reverse Transcriptase system with Oligo(dT) primers (Thermo Fisher Scientific). For real-time reactions, 10 μl of Sensifast qPCR Master Mix (Bioline, London, UK) was mixed with 100 mM forward and reverse primers and 2 μl of cDNA. Samples were analyzed in an ABI 7300 real-time PCR system (Applied Biosystems) with an initial step of 3 minutes at 95°C for polymerase activation, followed by 40 cycles at 95°C for 5 seconds for denaturation, and 60°C for 15 seconds for annealing/extension.333. Results were analyzed against human GAPDH as a housekeeping gene and expressed using the ΔΔCT method (Pfaffl, 2001). The sequences of primers can be found in Table 1. The results were expressed as the mean ± SD. The significance of differences was evaluated using ANOVA followed by Dunnett’s *post-hoc* test as indicated.

**Table 1:** Sequences of primers used in RT-qPCR

Gene	Forward primer	Reverse primer
TLR2	TCGGAGTTCTCCCAGTGTTTG	GCAGTGAAAGAGCAATGGGC
TLR4	GGTCAGACGGTGATAGCGAG	TTTAGGGCCAAGTCTCCACG
TLR7	TCCATGCCATCAAGAAAGTTGA	GTCTGTGCAGTCCACGATCA
TLR9	CAGCATGGGTTTCTGCCG	GGGCAGTTCCACTTGAGGTT
NOD1	CCTGGTGGCCAAGTGATTGTA	CCAAGCCTGCGATTCCCATA
NOD2	ATCCGGAGCCTGTACGAGAT	CGCGCAAATACAGAGCCTTG
IL-1β	CTTCGAGGCACAAGGCACAA	CTGGAAGGAGCACTTCATCTGT
IL6	ACCCCCAATAAATATAGGACTGGA	CGAAGGCGCTTGTGGAGAA
IL-12α	GCTCCAGAAGGCCAGACAAA	GCCAGAGCCTAAGACCTCAC
IFN-ɣ	TGGAAAGAGGAGAGTGACAGA	CTGTTTTAGCTGCTGGCGAC
IL-10	CGAGATGCCTTCAGCAGAGT	GGCAACCCAGGTAACCCTTA
TGFβ	TACCTGAACCCGTGTTGCTC	CCGGTAGTGAACCCGTTGAT
IL-17	TGGAATCTCCACCGCAATGA	GCTGGATGGGGACAGAGTTC
GAPDH	AACAGCGACACCCACTCCTC	GGAGGGGAGATTCAGTGTGGT

## Results

### *Trypanosoma cruzi* changes the gene expression profile in HPE

The effect of the parasite on placental tissue was assayed in HPE after challenges with a low (10^5^ parasites/ml) or a high (10^6^ parasites/ml) concentration of trypomastigotes for 2 or 24 h.

**Fig. 1:**
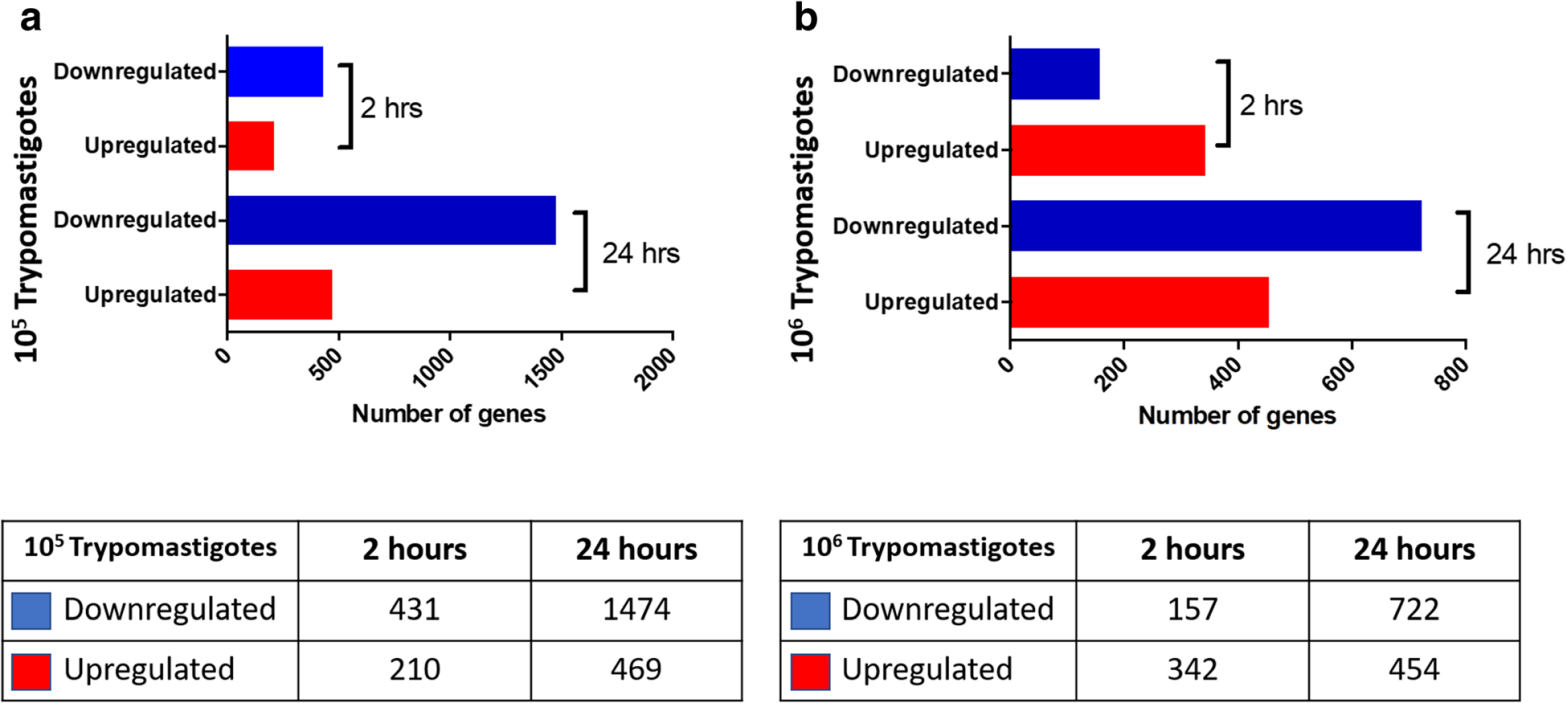
Differential gene expression in *Trypanosoma cruzi* infected HPCVE at 2 and 24 h compared to not-infected control samples. HPCVE were challenged with 10^5^ (**a**) or 10^6^ (**b**) trypomastigotes/ml. Blue bars indicate downregulated genes and red bars indicate upregulated genes. Inset table shows the number of differentially expressed genes for each condition. (≥ 2-fold, *P* ≤ 0.05)

Total RNA extracted from infected and non-infected control HPE, was labeled and hybridized to a Human GE 60K Microarray, which allows the evaluation of the gene expression profile of 26,083 different human genes. Genes showing at least a 2-fold change in their expression and a 95% probability of being differentially expressed (*P* ≤ 0.05) were significantly regulated during parasite challenge. Figure 1 shows the total number of significant differentially expressed genes between infected and non-infected control HPE. A low parasite concentration induces the downregulation of 431 and 1474 genes as well as the upregulation of 210 and 469 genes after 2 and 24 h of parasite challenge, respectively (Fig. 1a). After a high parasite concentration challenge, 157 and 722 genes were downregulated, and 342 and 454 were upregulated after 2 and 24 h, respectively (Fig. 1b). Major changes occurred after 24 h of parasite challenge with the lowest parasite concentration. A selection of the most upregulated and downregulated genes (fold change range between 34.70 and 71.43) is shown in Table 2. Among the most upregulated genes are those involved in immune response such as *CXCL9*, *TLR-7*, *TLR-8*, *CD46*, *C1qTNF3*, *HLA-DQB1* and *CCL20*; genes involved in extracellular matrix (ECM) remodeling (*ADAM12*, *ADAMTSL3*, *MMP10*) and related to pregnancy. The list of most downregulated genes also includes genes related to immunity such as *LBP*, *CD14*, *DCD* and *IL-6*.

**Table 2:** Upregulated and downregulated genes with fold change (FC) ≥ 20 at 2 and 24 h post-infection

Gene ID	Description	FC	Gene ID	Description	FC
2 h					
Upregulated 10^5^ trypomastigotes			Downregulated 10^5^ trypomastigotes		
LDOC1	Leucine zipper, downregulated in cancer 1	71.43	SLC9B1	Solute carrier family 9, subfamily B (NHA1, cation proton antiporter 1), member 1	65.64
ADAM12	ADAM metallopeptidase domain 12	54.99	LBP	Lipopolysaccharide binding protein	60.23
PNMT	Phenylethanolamine N-methyltransferase	51.74	MEDAG	Mesenteric estrogen-dependent adipogenesis	52.12
ADORA3	Adenosine A3 receptor	48.44	SYT1	Synaptotagmin I	49.30
GH2	Growth hormone 2	44.96	RGS7BP	Regulator of G-protein signaling 7 binding protein	49.08
PCDHB13	Protocadherin beta 13	44.71	MT1H	Metallothionein 1H	47.18
CXCL9	Chemokine (C-X-C motif) ligand 9	26.61	CAPN8	Calpain 8	46.86
TLR7	Toll-like receptor 7	23.52	DCD	Dermcidin	42.36
TLR8	Toll-like receptor 8	21.74	HTR5A	5-hydroxytryptamine (serotonin) receptor 5A, G protein-coupled	42.25
CD46	CD46 molecule, complement regulatory protein	21.48	KALRN	Kalirin, RhoGEF kinase	42.01
C1QTNF3	C1q and tumor necrosis factor related protein 3	21.41	CDH18	Cadherin 18, type 2	35.52
Upregulated 10^6^ trypomastigotes			Downregulated 10^6^ trypomastigotes		
LOC101060810	Zinc finger protein 98-like	68.80	SNRPG	Small nuclear ribonucleoprotein polypeptide G	73.40
LMOD1	Leiomodin 1 (smooth muscle)	62.19	TUBA1C	Tubulin, alpha 1c	71.97
SPIN4	Spindlin family, member 4	53.45	TSC22D1	TSC22 domain family, member 1	71.15
LINC00551	Long intergenic non-protein coding RNA 551	52.98	CD14	CD14 molecule	70.98
PSG3	Pregnancy specific beta-1-glycoprotein 3	47.68	MBNL2	Muscleblind-like splicing regulator 2	64.74
PSG8	Pregnancy specific beta-1-glycoprotein 8	45.17	FUT3	Fucosyltransferase 3 (galactoside 3(4)-L-fucosyltransferase. Lewis blood group)	63.73
PSPHP1	Phosphoserine phosphatase pseudogene 1	44.87	GCGR	Glucagon receptor	62.52
PSG1	Pregnancy specific beta-1-glycoprotein 1	42.74	IL6	Interleukin 6 (interferon, beta 2)	62.25
PCDHB13	Protocadherin beta 13	41.77	NOG	Noggin	60.99
HULC	Hepatocellular carcinoma upregulated long non-coding RNA	40.94	SBSN	Suprabasin	60.01
ADAMTSL3	ADAMTS-like 3	34.70	FAM89A	Family with sequence similarity 89, member A	57.57
24 h					
Upregulated 10^5^ trypomatigotes			Downregulated 10^5^ trypomastigotes		
GCGR	Glucagon receptor	26.78	GEMIN2	Gem (nuclear organelle) associated protein 2	66.26
CHRDL2	Chordin-like 2	26.46	GUCA1A	Guanylate cyclase activator 1A (retina)	50.37
TAC3	Tachykinin 3	23.82	LOC340515	Uncharacterized LOC340515	47.71
STC1	Stanniocalcin 1	21.80	LINC00200	Long intergenic non-protein coding RNA 200	42.88
SLC43A3	Solute carrier family 43, member 3	21.60	FAM182B	Family with sequence similarity 182, member B	41.35
PENK	Proenkephalin	21.06	SEC14L4	SEC14-like 4 (*S. cerevisiae*)	38.81
SLC44A4	Solute carrier family 44, member 4	21.02	CLEC6A	C-type lectin domain family 6, member A	38.42
HLA-DQB1	Major histocompatibility complex, class II, DQ beta 1	20.99	SNORA65	Small nucleolar RNA, H/ACA box 65	36.94
ANTXR1	Anthrax toxin receptor 1	20.21	LOC541473	FK506 binding protein 6, 36kDa pseudogene	36.81
SBSN	Suprabasin	20.18	AKR7L	Aldo-keto reductase family 7-like	36.51
Upregulated 10^6^ trypomastigotes			Downregulated 10^6^ trypomastigotes		
MMP10	Matrix metallopeptidase 10 (stromelysin 2)	105.67	DNMT3L	DNA (cytosine-5-)-methyltransferase 3-like	63.39
CSH2	Chorionic somatomammotropin hormone 2	68.94	KLRG2	Killer cell lectin-like receptor subfamily G, member 2	46.45
GH2	Growth hormone 2	68.66	MS4A6A	Membrane-spanning 4-domains. subfamily A, member 6A	40.49
CCL20	Chemokine (C-C motif) ligand 20	58.87	TMEM45B	Transmembrane protein 45B	40.27
ENO2	Enolase 2 (gamma, neuronal)	53.70	IGFBP1	Insulin-like growth factor binding protein 1	39.99
SOD2	Superoxide dismutase 2, mitochondrial	53.42	PSPHP1	Phosphoserine phosphatase pseudogene 1	39.83
PCDHB13	Protocadherin beta 13	53.33	TPTE2P3	Transmembrane phosphoinositide 3-phosphatase and Tensin homolog 2 pseudogene 3	39.87
SELE	Selectin E	52.93	MNDA	myeloid cell nuclear differentiation antigen	38.99
ABO	ABO blood group (transferase A, alpha 1-3-N-acetylgalactosaminyltransferase; transferase B, alpha 1-3-galactosyltransferase)	51.78	FGF14-AS2	FGF14 antisense RNA 2	38.98
STC1	Stanniocalcin 1	51.40	PTX3	Pentraxin 3, long	38.59

**Fig. 2:**
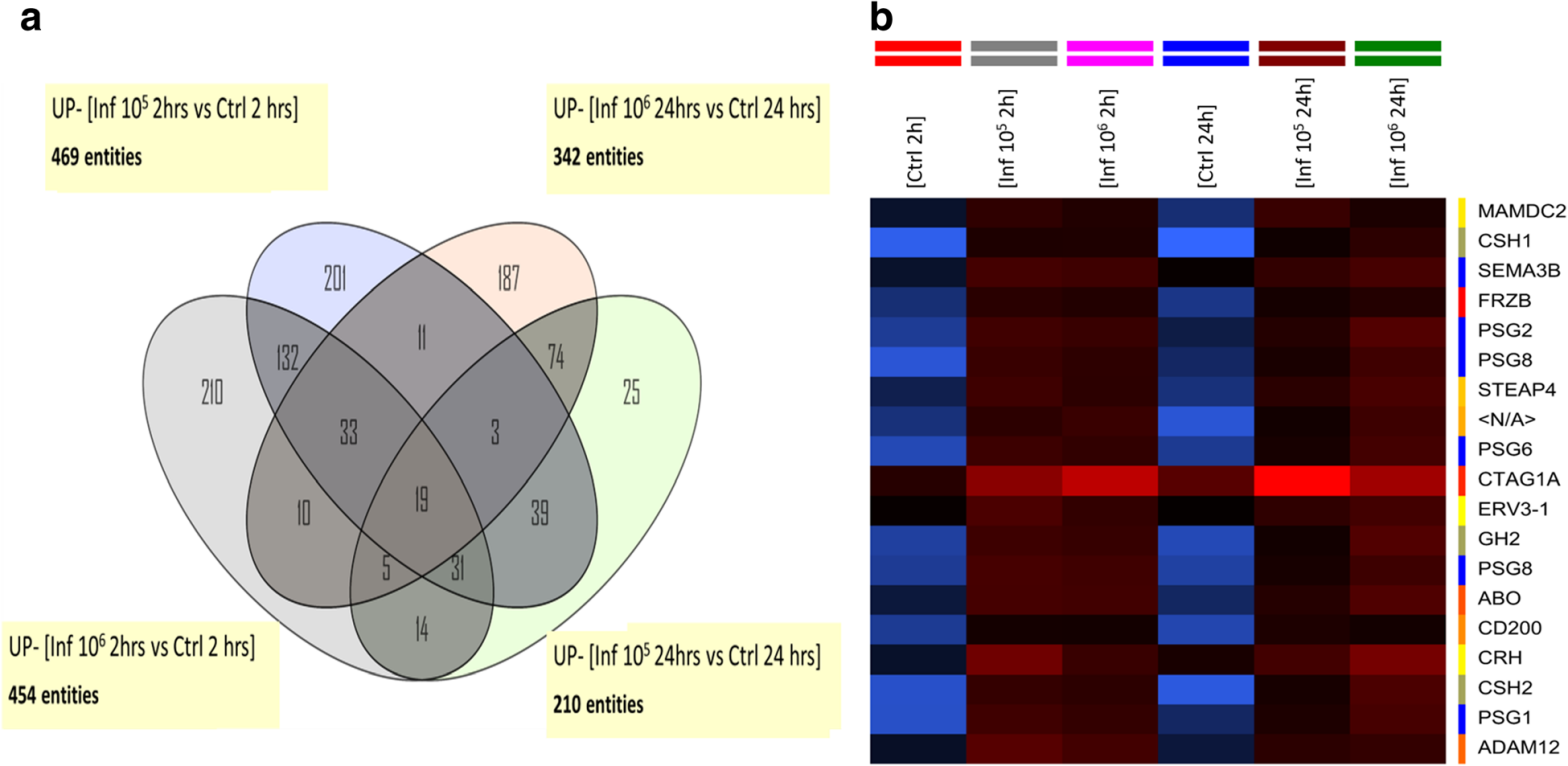
Venn diagrams comparing common differentially upregulated genes. HPCVE were incubated for 2 and 24 h with 10^5^ or 10^6^ *T. cruzi* trypomastigotes/ml. All samples were compared to the respective uninfected control. The diagram in **a** shows the upregulated genes at both parasite concentrations and incubation times, **b** corresponds to the heatmap of the differentially expressed genes in the central intersection

**Fig. 3:**
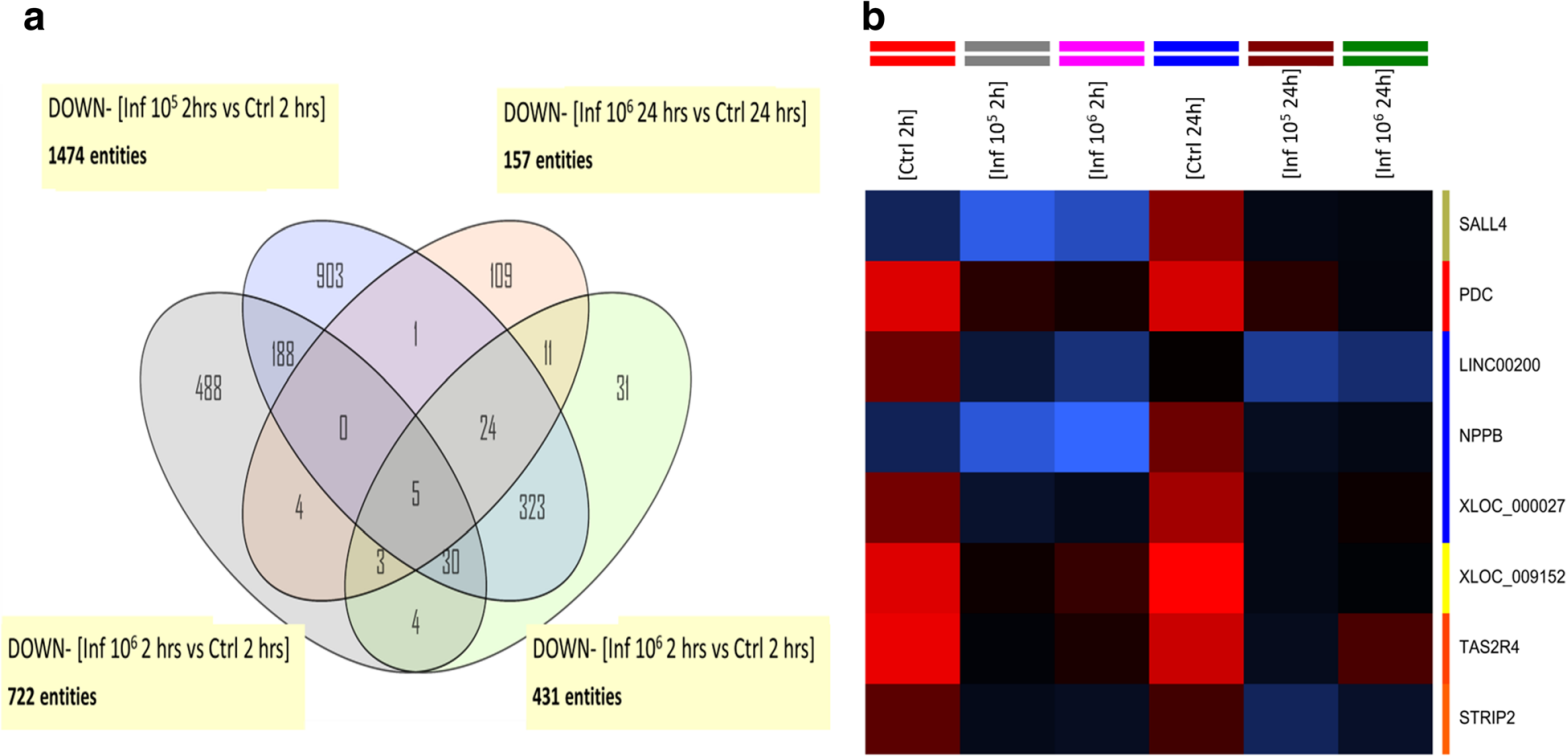
Venn diagrams comparing common differentially downregulated genes. HPCVE were incubated for 2 and 24 h with 10^5^ or 10^6^ *T. cruzi* trypomastigotes/ml. All samples were compared to the respective uninfected control. The diagram in **a** shows the downregulated genes at both parasite concentrations and incubation times, **b** corresponds to the heatmap of the differentially expressed genes in the central intersection

The Venn diagrams in Fig. 2a show that 19 genes are upregulated in the four different experimental conditions compared to control non-infected samples, which are also shown in the corresponding heatmap (Fig. 2b) listed in Table 3. Contrarily, only 5 genes are downregulated in the same conditions (Fig. 3a, b), which are listed in Table 3. Most of the upregulated genes are related to pregnancy processes.

**Table 3:** Upregulated and downregulated genes in the four different experimental conditions compared to control non-infected samples

Gene symbol	Description
Upregulated genes	
MAMDC2	*Homo sapiens* MAM domain containing 2 (MAMDC2), mRNA [NM_153267]
CSH1	*Homo sapiens* chorionic somatomammotropin hormone 1 (placental lactogen) (CSH1), mRNA [NM_001317]
SEMA3B	*Homo sapiens* sema domain, immunoglobulin domain (Ig), short basic domain, secreted, (semaphorin) 3B (SEMA3B), transcript variant 1, mRNA [NM_004636]
FRZB	*Homo sapiens* frizzled-related protein (FRZB), mRNA [NM_001463]
PSG2	*Homo sapiens* pregnancy specific beta-1-glycoprotein 2 (PSG2), mRNA [NM_031246]
PSG8	*Homo sapiens* pregnancy specific beta-1-glycoprotein 8 (PSG8), transcript variant 1, mRNA [NM_182707]
STEAP4	*Homo sapiens* STEAP family member 4 (STEAP4), transcript variant 2, mRNA [NM_001205315]
	Uncharacterized protein [Source: UniProtKB/TrEMBL; Acc: B8ZZY5] [ENST00000409490]
PSG6	*Homo sapiens* pregnancy specific beta-1-glycoprotein 6 (PSG6), transcript variant 1, mRNA [NM_002782]
CTAG1A	*Homo sapiens* cancer/testis antigen 1A (CTAG1A), mRNA [NM_139250]
ERV3-1	*Homo sapiens* endogenous retrovirus group 3, member 1 (ERV3-1), mRNA [NM_001007253]
GH2	*Homo sapiens* growth hormone 2 (GH2), transcript variant 3, mRNA [NM_022558]
PSG8	*Homo sapiens* pregnancy specific beta-1-glycoprotein 8 (PSG8), transcript variant 1, mRNA [NM_182707]
ABO	ABO blood group (transferase A, alpha 1-3-N-acetylgalactosaminyltransferase; transferase B, alpha 1-3-galactosyltransferase) [Source: HGNC Symbol; Acc:79] [ENST00000319878]
CD200	*Homo sapiens* CD200 molecule (CD200), transcript variant 2, mRNA [NM_001004196]
CRH	*Homo sapiens* corticotropin releasing hormone (CRH), mRNA [NM_000756]
CSH2	*Homo sapiens* chorionic somatomammotropin hormone 2 (CSH2), transcript variant 2, mRNA [NM_022644]
PSG1	*Homo sapiens* pregnancy specific beta-1-glycoprotein 1 (PSG1), transcript variant 1, mRNA [NM_006905]
ADAM12	*Homo sapiens* ADAM metallopeptidase domain 12 (ADAM12), transcript variant 2, mRNA [NM_021641]
Downregulated genes	
SALL4	*Homo sapiens* spalt-like transcription factor 4 (SALL4), mRNA [NM_020436]
LINC00200	*Homo sapiens* long intergenic non-protein coding RNA 200 (LINC00200), long non-coding RNA [NR_015376]
NPPB	*Homo sapiens* natriuretic peptide B (NPPB), mRNA [NM_002521]
TAS2R4	*Homo sapiens* taste receptor, type 2, member 4 (TAS2R4), mRNA [NM_016944]
STRIP2	*Homo sapiens* striatin interacting protein 2 (STRIP2), transcript variant 1, mRNA [NM_020704]
FRZB	*Homo sapiens* frizzled-related protein (FRZB), mRNA [NM_001463]

### *Trypanosoma cruzi* alters a wide range of biological processes in HPE

**Fig. 4:**
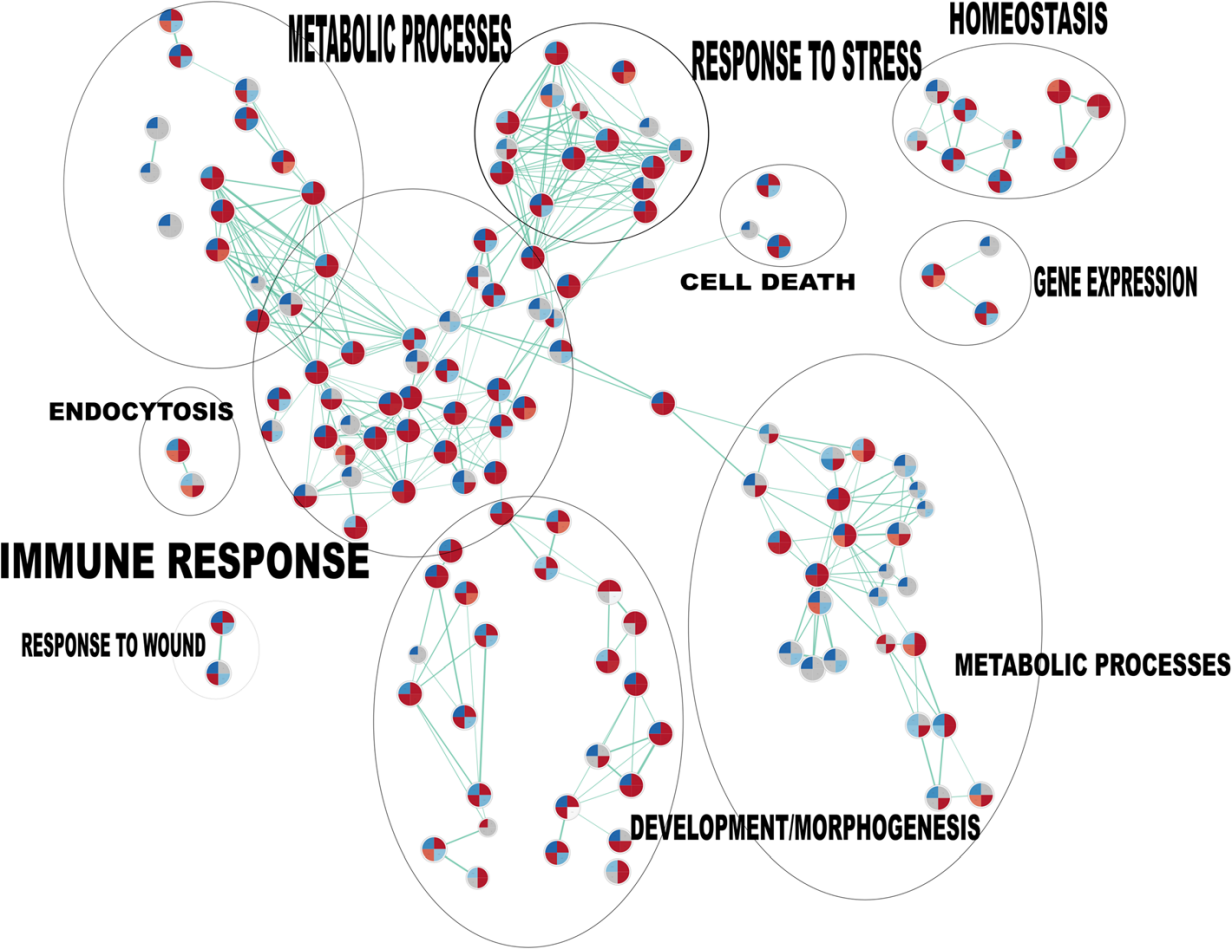
Enrichment map of gene sets of biological processes pathways. HPCVE were incubated for 2 and 24 h with 10^5^ or 10^6^ *T. cruzi* trypomastigotes/ml. Genes with fold changes ≥ 2 (*P* ≤ 0.05) were considered for the analysis. Gene set analysis (GSEA) was performed based in pathways from GO biological processes. Nodes correspond to gene sets and connecting lines to overlapping member genes between nodes. Divided circles represent predicted pathways, each segment of the circle represents a different experimental group according to attached legend and colors depict upregulated (red) or downregulated (blue) genes. Clusters grouped by biological function were manually labeled

GO and pathway analysis were performed using GeneSpringGX 13.0 software (Agilent Technologies), comparing the different experimental conditions described above. Our results indicate that a wide range of biological processes are altered at the different conditions in presence of the parasite (Table 4). The different biological processes detected include immune response, pregnancy related processes and signaling. In order to understand the relationships between those biological processes, we performed a GSEA analysis of gene sets at different times and parasite load challenges. We analyzed biological processes pathways based on gene ontology results (Fig. 4). The biggest cluster is composed of pathways related with immune response, followed by development morphogenesis cluster, regulation of metabolic processes and signal transduction genes, metabolic processes, homeostasis, response to stimulus, cell death and endocytosis (Fig. 4).

**Table 4:** Biological processes predicted to be modulated during *T. cruzi* infection

GO Accession	GO term	No. of genes/condition			
		10^5^ 2 h	10^6^ 2 h	10^5^ 24 h	10^6^ 24 h
Upregulated biological processes					
GO:0022414	Reproductive process	45	42	45	114
GO:0032501|GO:0050874	Multicellular organismal process	191	189	326	550
GO:0050896|GO:0051869	Response to stimulus	208	196	728	623
GO:0051704|GO:0051706	Multi-organism process	56	58	204	197
GO:0065007	Biological regulation	269	255	977	836
GO:0002376	Immune system process	39	36	236	215
GO:0009987|GO:0008151|GO:0050875	Cellular process	346	334	1161	987
GO:0022610	Biological adhesion	35	23	134	99
GO:0023052|GO:0023046	Signaling	141	132	543	464
GO:0044699	Single-organism process	342	0	1125	972
GO:0051179	Localization	125	0	415	372
GO:0071840|GO:0071841	Cellular component organization or biogenesis	105	0	427	307
GO:0008152	Metabolic process	238	0	0	629
GO:0032502	Developmental process	144	0	0	434
Downregulated biological processes					
GO:0009987|GO:0008151|GO:0050875	Cellular processes	902	681	937	648
GO:0023052|GO:0023046	Signaling	388	0	376	0
GO:0032501|GO:0050874	Multicellular organismal process	516	0	490	0
GO:0044699	Single-organism processes	903	620	880	615
GO:0050896|GO:0051869	Response to stimulus	547	371	507	328
GO:0065007	Biological regulation	697	505	734	487
GO:0071840|GO:0071841	Cellular component organization or biogenesis	0	240	0	248
GO:0022414	Reproductive processes	0	0	106	0
GO:0051179	Localization	0	0	275	0
GO:0051704|GO:0051706	Multi-organism processes	0	0	123	0

Several gene sets grouped within the main cluster (immune response), are enriched at 2 or 24 h post-infection after parasite challenges of 10^5^ trypomastigotes/ml but not of 10^6^ parasites. Thus, immune system process pathways are positively regulated against 10^5^ parasites at 2 h (NES: 1.57; FDR q-value = 0.0013) and 24 h (NES: 2.75; FDR q-value = 0.0018), downregulated with 10^6^ trypomastigotes/ml at 2 h (NES: -2.1; FDR q-value = 0.0087) but not at 24 h (NES: 1.44; FDR q-value = 0.18). Regulation of immune response is positively regulated with 10^5^ parasites at 2 h (NES: 1,50; FDR q-value = 0.022) and 24 h (NES: 1.98; FDR q-value = 0.01) but negatively with 10^6^ parasites at 2 h (NES: -1,77; FDR q-value = 0.067) and without significant changes at 24 h (NES: -0,83; FDR q-value not significant).

Gene Ontology process regulation of cytokine production is upregulated 2 h post-infection (NES: 1.36; FDR q-value = 0.023) but not in the other experimental groups. The inflammatory response pathway is upregulated 24 h post-infection against 10^5^ trypomastigotes (NES: 1.65; FDR q-value = 0.049) but downregulated with 10^6^ parasites 2 h post-infection (NES: 1.99; FDR q-value = 0.023). Amongst the changes of pathways related to other processes, it can be highlighted the upregulation of cell proliferation pathway 24 h post-infection (NES: 2.18; FDR q-value = 0.003) and cellular response to hormone stimulus (2 h 10^5^ Trypos NES: 2.53; FDR q-value = 0.005).

To understand the nature of the interaction between upregulated or downregulated genes, we performed a gene interaction analysis using the GeneMANIA plug-in for Cytoscape 3.0 software [24, 25]. For each experimental condition, we analyzed co-expression, co-localization, physical interactions, genetic interactions shared protein domains and pathways amongst all the differentially expressed genes (Fc ≥ 2) in both upregulated or downregulated gene lists. The relative weight of each process between all interacting genes is depicted in Table 5. In all experimental conditions, co-expression (a category where two genes have similar expression levels) is the predominating interaction, co-localization (genes expressed in the same tissue) the second in the upregulated conditions; however, in downregulated groups physical interactions (when two gene products are found to interact in protein-protein interaction studies) is the second most common interaction. Shared protein domains, genetic and pathways interaction represent marginal interactions in all groups. A circular layout of the interaction network for each is shown in Additional file 1: Figure S1.

**Table 5:** Relative weight of gene interactions in *T. cruzi*-infected HPE

Network group	2 h/10^5^ Up	2 h/10^5^ Down	2 h/10^6^ Up	2 h/10^6^ Down	24 h/10^5^ Up	24 h/10^5^ Down	24 h/10^6^ Up	24 h/10^6^ Down
Co-expression	72	61	64	42	75	66	75	75
Co-localization	13	7	18	9	11	6	14	3
Physical interactions	7	14	11	31	6	17	6	9
Genetic interactions	4	0	2	4	3	0	0	1
Shared protein domains	0	1	1	3	0	1	1	0
Pathway	0	1	1	3	0	0	1	7
Others	4	16	3	8	5	10	3	5

*Abbreviations*: Up, upregulated; Down, downregulated; H, hours of HPE incubation with the parasite; 10^5^, challenge with 10^5^ parasites/ml; 10^6^, challenge with 10^6^ parasites/ml

### *Trypanosoma cruzi* induces differential expression of pathogen pattern recognition receptors in HPE

**Fig. 5:**
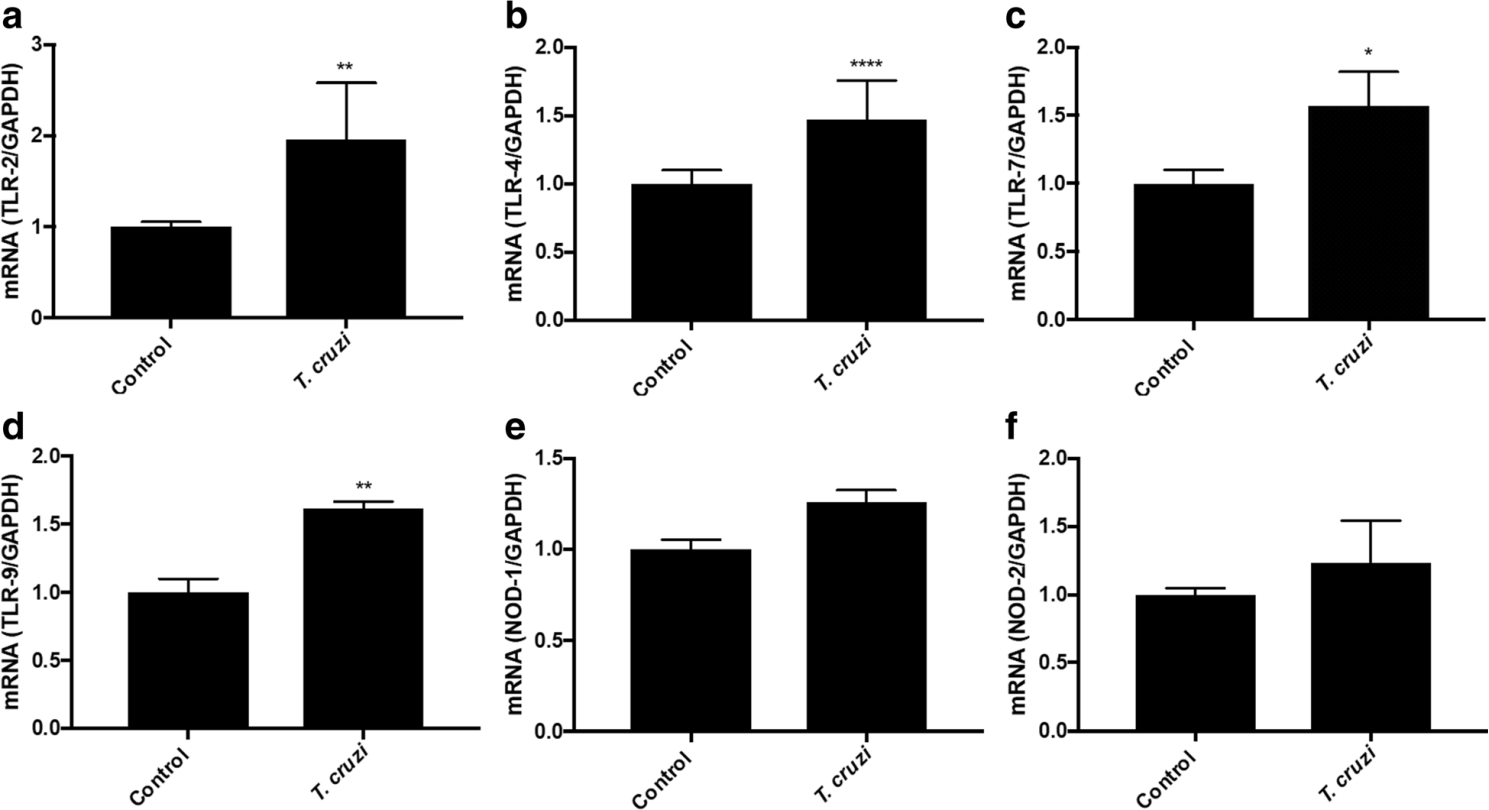
*Trypanosoma cruzi* induce differential expression of pathogen pattern recognition receptors in HPE. Explants were co-incubated with 10^5^ *T. cruzi* trypomastigotes/ml for 2 h and the expression of TLR-2 (**a**), TLR-4 (**b**), TLR-7 (**c**), TLR-9 (**d**), NOD-1 (**e**) and NOD-2 (**f**) were assayed. Samples were processed for RT-qPCR. The sequences of primers can be found in Table 1. The significance of differences was evaluated using Student’s t-tests for paired data. *P ≤ 0.05, **P ≤ 0.01, ****P ≤ 0.0001

Based on the results of the microarray analysis showing an activation of local immune processes, we decided to validate by RT-qPCR PRR activation and cytokine production. Explants were co-incubated with 10^5^ *T. cruzi* trypomastigotes for 2 h and the expression of *Toll*-like receptors (TLRs) and NOD-like receptors (NLRs) was assayed. *Trypanosoma cruzi* trypomastigotes induce statistically significant increases of TLR-2 (96.13 ± 61.6%; *F*_(2, 9)_ = 5.409; *P* ≤ 0.01, Fig. 5a), TLR-4 (47.56 ± 27.99%; *F*_(2, 45)_ = 2.173; *P* ≤ 0.0001, Fig. 5b), TLR-7 (57.29 ± 24.59%; *F*_(2, 2)_ = 6.048; *P* ≤ 0.05, Fig. 5c) and TLR-9 (61.56 ± 5.11%; *F*_(2, 6)_ = 1.630; *P* ≤ 0.01, Fig. 5d) expression compared to non-infected HPE. However, *T. cruzi* does not increase significantly NOD-1 and NOD-2 receptors (Fig. 5d, e).

### *Trypanosoma cruzi* increases pro-inflammatory and immune-modulating cytokines in HPE

**Fig. 6:**
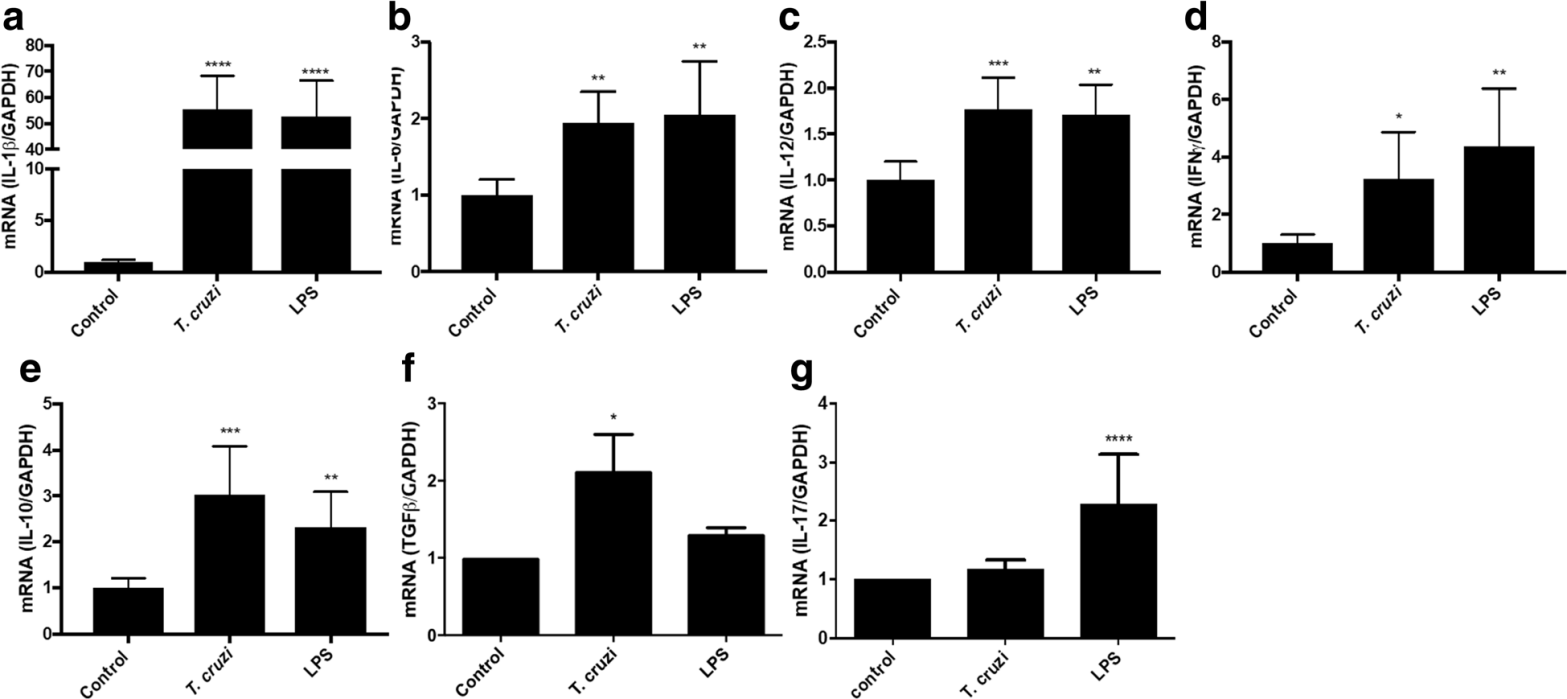
*Trypanosoma cruzi* increases pro-inflammatory and immune-modulating cytokines in HPE. HPE were co-incubated with 10^5^ *T. cruzi* trypomastigotes/ml for 2 h as well as in the presence and absence of LPS as positive control. Expression of IL-1β (**a**), IL-6 (**b**), IL-12α (**c**), IFNγ (**d**), IL-10 (**e**), TGF β (**f**) and IL-17 (**g**) was assayed. Samples were processed for RT-qPCR. The sequences of primers can be found in Table 1. The significance of differences was evaluated using Student’s t-tests for paired data. *P ≤ 0.05, **P ≤ 0.01, ***P ≤ 0.001, ****P ≤ 0.0001

HPE were co-incubated with 10^5^ *T. cruzi* trypomastigotes for 2 h as well as in the presence and absence of LPS as positive controls. *T. cruzi* induces significant increases of the pro-inflammatory cytokines IL-1β (5542.55 ± 1090.11%; *F*_(2, 19)_ = 64.91; *P* ≤ 0.0001, Fig. 6a), IL-6 (94.70 ± 40.38%; *F*_(2, 15)_ = 8.849; *P* ≤ 0.01, Fig. 6b), IL-12α (77.08 ± 33.01%; *F*_(2, 19)_ = 64.91; *F*_(2, 15)_ = 16.13; *P* ≤ 0.01, Fig. 6c), IFNγ (329.29 ± 162.22%; *F*_(2, 17)_ = 7.729; *P* ≤ 0.05, Fig. 6d) and of the immune-modulating cytokines IL-10 (303.34 ± 104.28%; *F*_(2, 20)_ = 14.87; *P* ≤ 0.001, Fig. 6e) and TGF β (329.29 ± 162.22%; *F*_(2, 11)_ = 1.684; *P* ≤ 0.05, Fig. 6f) but not of IL-17 (Fig. 6g).

## Discussion

The interaction between the host and pathogens, including *T. cruzi*, is the most important factor in determining whether an infection is successful. Host-parasite interaction includes invasion of the host through primary barriers (such as the placental barrier), evasion of host defenses, pathogen replication in the host, and immunological capacity of the host to control or eliminate the pathogen [26]. Importantly, infected organisms are capable of sensing the intrusion by pathogens and react by triggering host defenses [17, 26]. On the other hand, the parasite is equipped with multiple tools to establish a long-term relationship with the infected host. Tissue infection in particular is relevant during disease progression. The presence of the parasite provokes tissue damage as well as immune and reparatory responses, which can lead to fibrosis and tissue dysfunction as observed in chagasic cardiomyopathy [27]. Considering the temporary existence of the placenta, the effect on parasite infection on this particular tissue is relevant to understand the physiopathology of congenital transmission in order to obtain tools for diagnosis, prognosis and treatment of the disease.

Previous studies about transcriptomics related to *T. cruzi* and Chagas disease have been focused on a single type cell response [10, 28] or on tissues or organs in animal models [24, 29] but not on human tissues. Here, we describe for the first time, the transcriptomics of an *ex vivo* human placental tissue model in response to challenges with the parasite.

As expected, *T. cruzi* modifies an ample range of biological processes during tissue invasion and infection. As described before, the parasite dramatically changes the gene expression in single cells [10]. However, in tissue and organ samples a more complex change in gene expression can be expected since they are composed of different cell types or tissues. For instance, in HPE epithelial cells derived from the trophoblast and fetal capillaries as well as fibroblasts and macrophages in the fetal connective tissue can be found, between others [3]. In addition, ECM components, that are synthetized by the resident cells are also present in tissue and organ samples [18, 19]. Similar results have been obtained in animal models, where important changes in murine myocardium metabolic pathways [29] and in murine placental response [24] are described. The increase of gene expression of proteases involved in ECM-remodeling (Table 2) is in concordance with our previous results showing that the parasite increased expression and activity of matrix metalloproteases (MMP-2 and MMP-9) in human placenta [19]. The profound changes in genes involved in signaling agrees with numerous previous studies that showed that *T. cruzi* activates or inhibits several signal transduction pathways [14, 28, 30].

The effect of *T. cruzi* on the immune system responses are particularly relevant since they are our main defense against the pathogen. The parasite dramatically changes host genes involved in the immune response. The expression of an important number of genes of innate immunity is increased. Thus, genes related to complement regulation and function such as CD46 and C1q are upregulated. We have previously shown, that during *ex vivo* infection of HPE, *T. cruzi* calreticulin (TcCRT) acts as a virulence factor since it binds maternal classical complement component C1q and increases parasite infectivity [31]. On the other hand, TLRs are also increased, particularly TLR-7 and TLR-8 which are increased over 20-fold. The validation experiments show that both mentioned TLRs as well as TLR-2, TLR-4 and TLR-9 are significantly increased (Fig. 5). However, TLR-2, the TLR whose inhibition increases parasite infection in HPE as well as parasite-induced tissue damage [17] showed no significant increase in the microarray analysis (Additional file 1: Figure S1).

A similar contradictory result was obtained with IL-6; a high parasite concentration decreases the expression of this cytokine more than 60-fold (Table 2). However, a low parasite concentration does not change IL-6 expression (Additional file 1: Figure S1). However, our RT-qPCR data show a significant increase of IL-6 (Fig. 5) that is in concordance with the increase of IL-6 protein in the culture media of HPE after parasite challenge in the same condition [17], suggesting regulation at post-transcriptional levels.

Another important group of genes that change their expression are those related to pregnancy. Most of the 19 upregulated genes in the four different experimental conditions compared to control samples are related to pregnancy processes (Table 3) and to the maintenance and development of the fetus such as pregnancy specific beta-1-glycoproteins, *GH2* (growth hormone 2), *CSH1* and *CSH2* (chorionic somatomammotropin hormone 1 and 2). Given that the placenta is the sole interface between mother and fetus and that this organ not only protects the fetus from infection but also regulates important metabolic and other physiological processes [32], it appears easily explainable that different pregnancy related processes are affected.

## Conclusions

*Trypanosoma cruzi* induces strong changes in genes involved in a wide range of pathways, especially those involved in immune response against infections.

## Additional file

Additional file 1: **Figure S1.** Interaction networks in differentially expressed genes from each experimental group. HPCVE were incubated during 2 and 24 h with 10^5^ or 10^6^ *T. cruzi* trypomastigotes. Interaction networks from differentially expressed genes (FC ≥ 2) compared with uninfected control with GeneMANIA function prediction service plug-in in Cytoscape software. Co-expression, co-localization, physical interactions, genetic interactions shared protein domains and pathways are shown and each color represents specific interactions according to legend. (PDF 707 kb)
