# Supplementary material for: Host-parasite interaction: changes in human placental gene expression induced by Trypanosoma cruzi
Source: Parasit Vectors. 2018 Aug 24;11:479. doi: 10.1186/s13071-018-2988-0 (PMC6109360; doi:10.1186/s13071-018-2988-0)
Supplement: Supplementary file 1 — Figure S1. Interaction networks in differentially expressed genes from each experimental group. HPCVE were incubated during 2 and 24 h with 105 or 106 T. cruzi trypomastigotes. Interaction networks from differentially expressed genes (FC ≥ 2) compared with uninfected control with GeneMANIA function prediction service plug-in in Cytoscape software. Co-expression, co-localization, physical interactions, genetic interactions shared protein domains and pathways are shown and each color represents specific interactions according to legend. (PDF 707 kb) [file 13071_2018_2988_MOESM1_ESM.pdf]

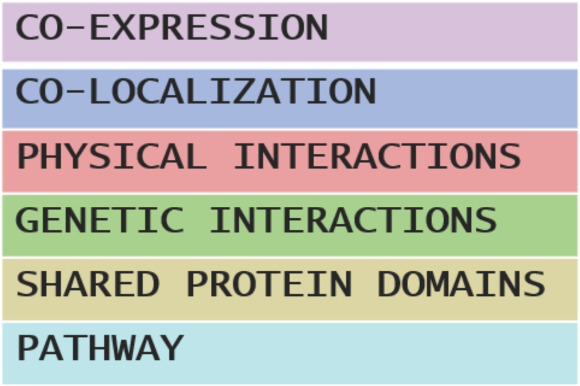

Supplementary Figure 1: Interaction networks in differentially expressed genes from each experimental group. HPCVE were incubated during 2 and 24 h with 10^5 or 10^6 *T. cruzi* trypomastigotes. Interaction networks from differentially expressed genes (FC≥2) compared with uninfected control with GeneMANIA function prediction service plug-in in Cytoscape software. Co-expression, co-localization, physical interactions, genetic interactions shared protein domains and pathways are shown and each color represents specific interactions according to legend.

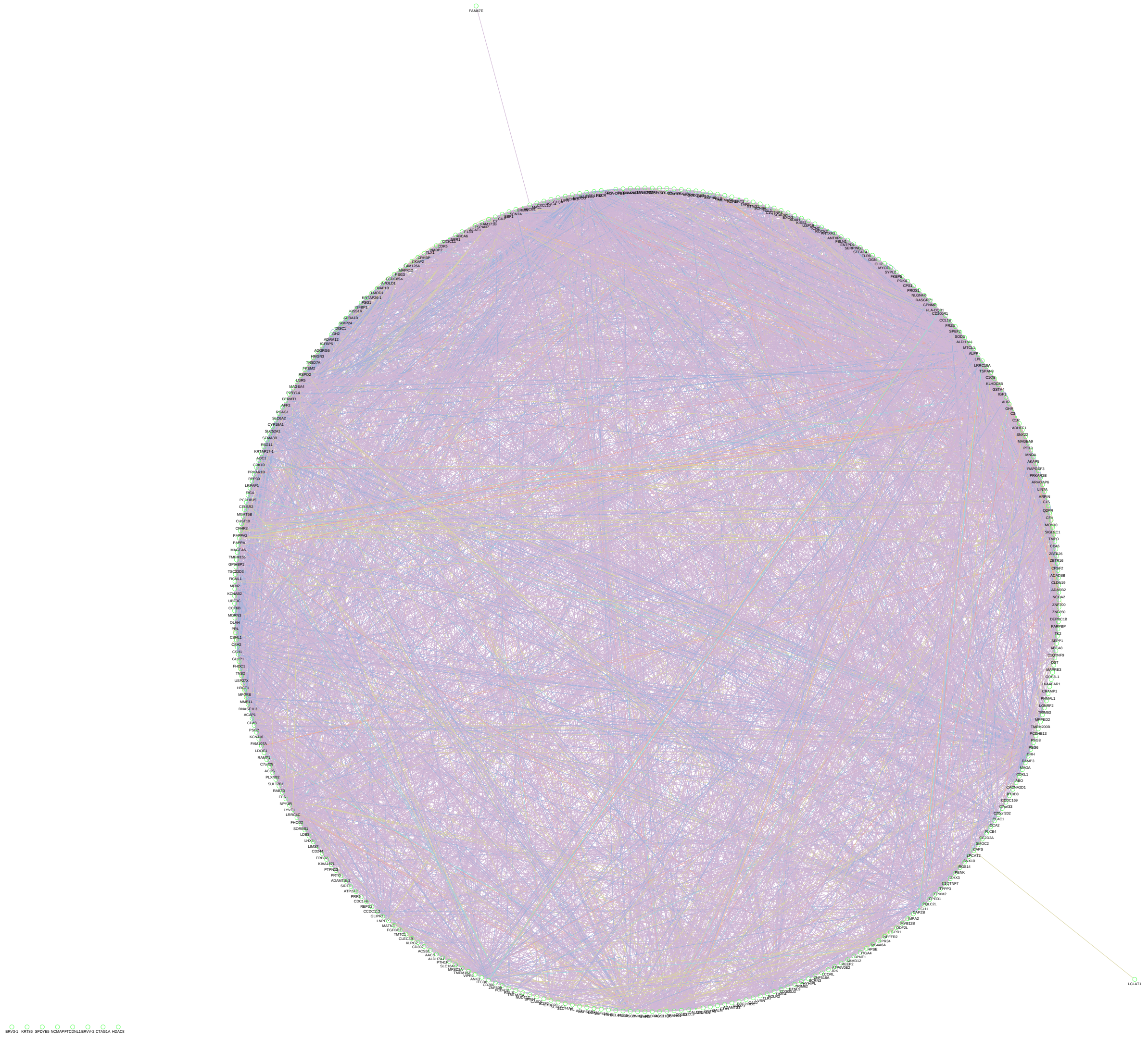

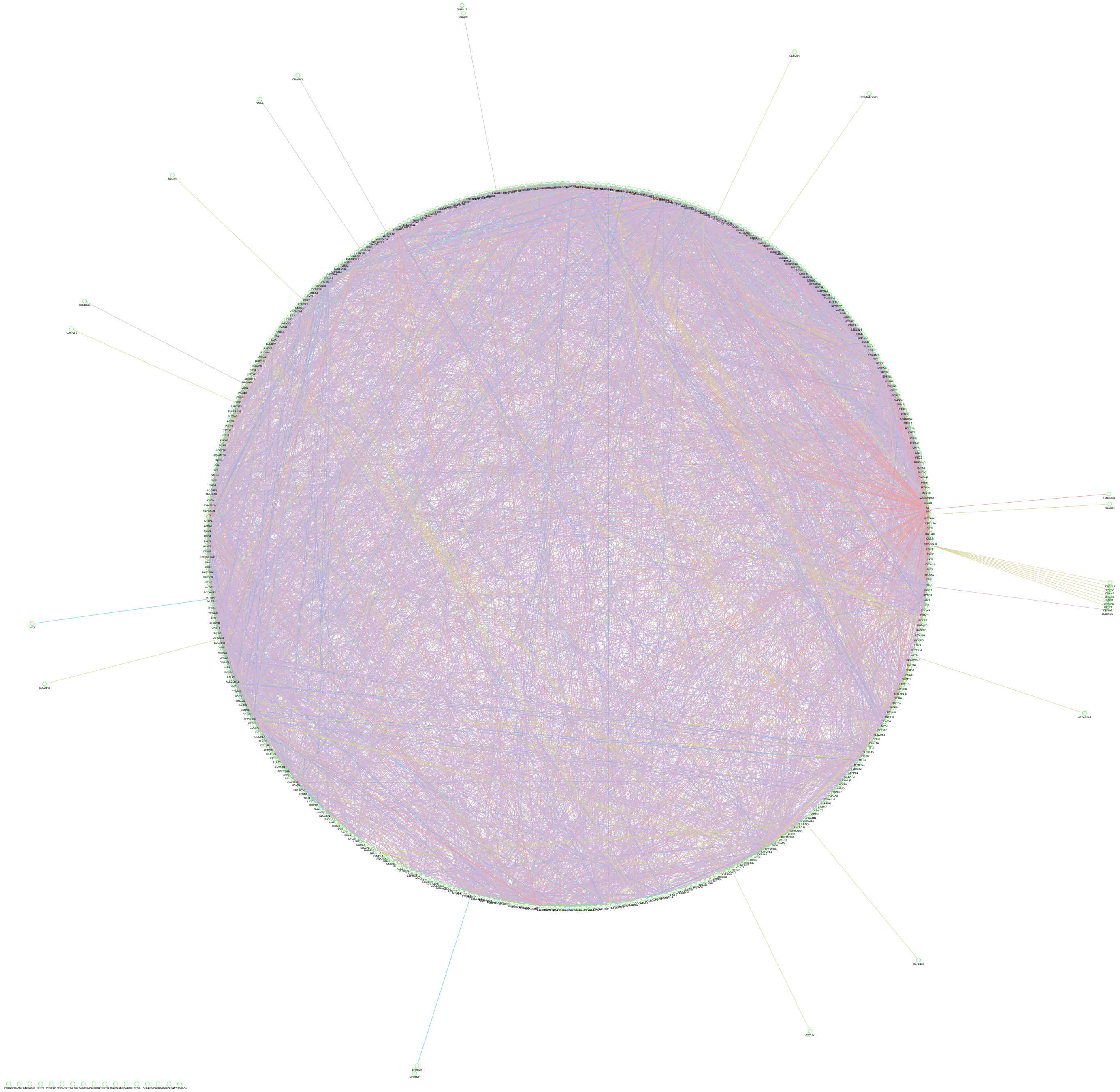

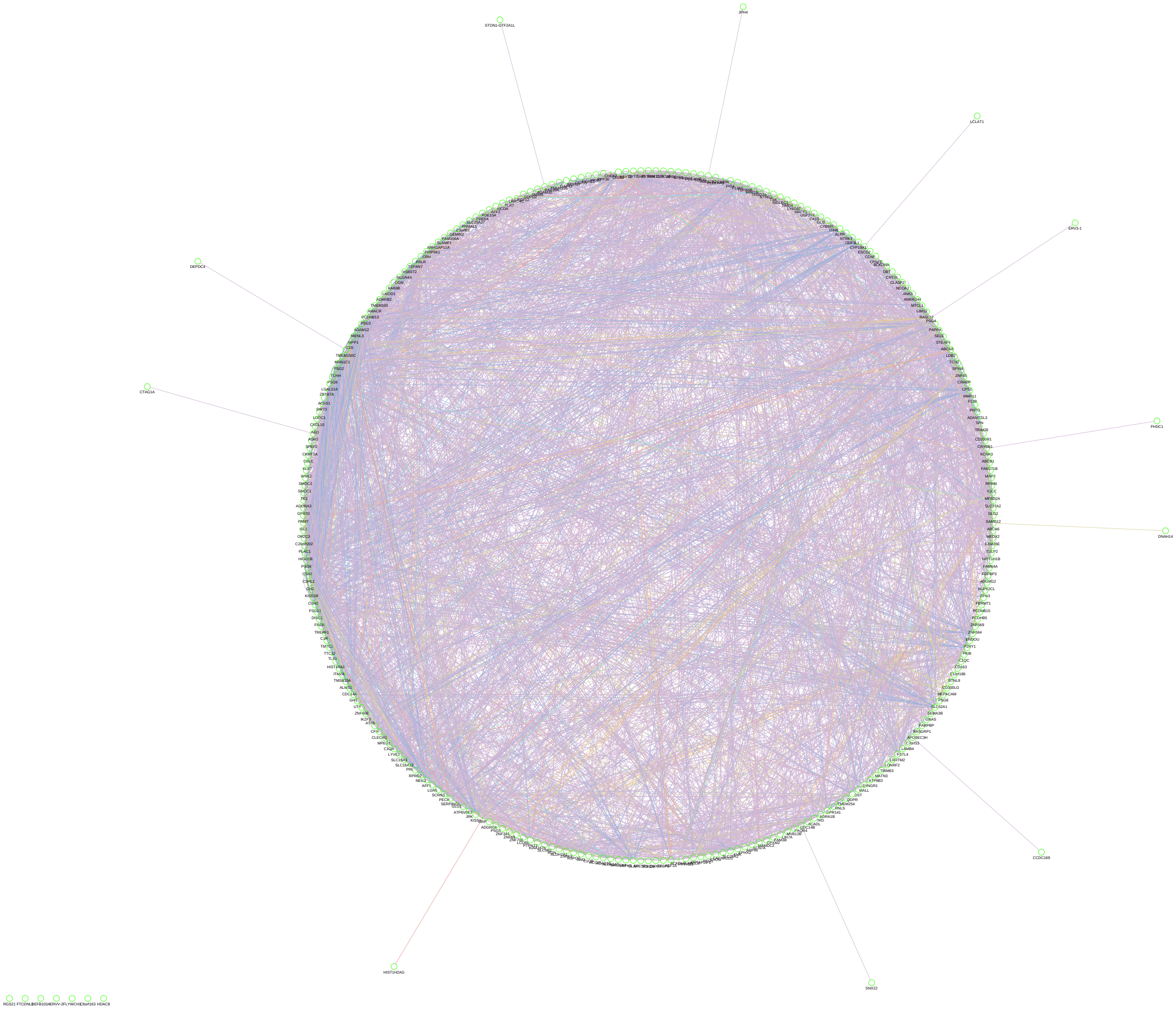

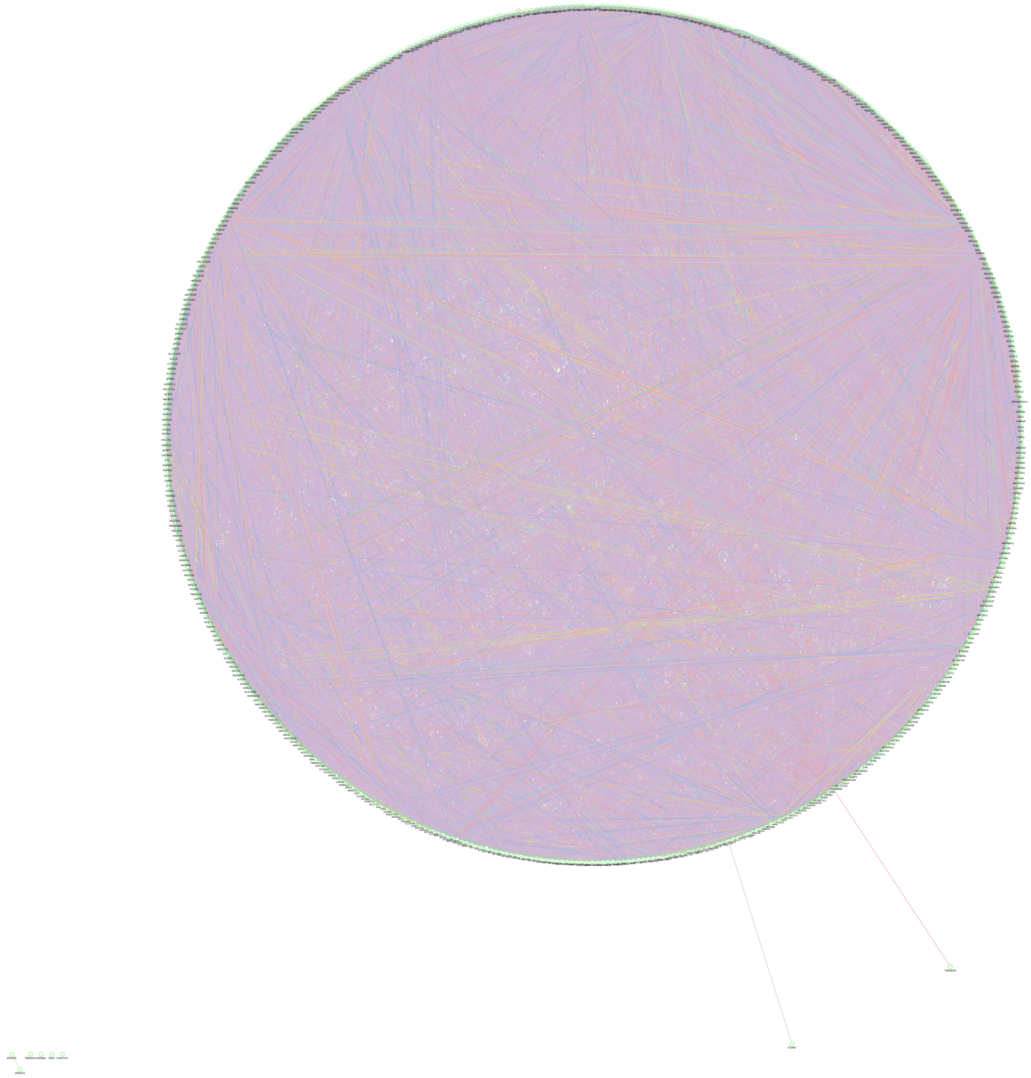

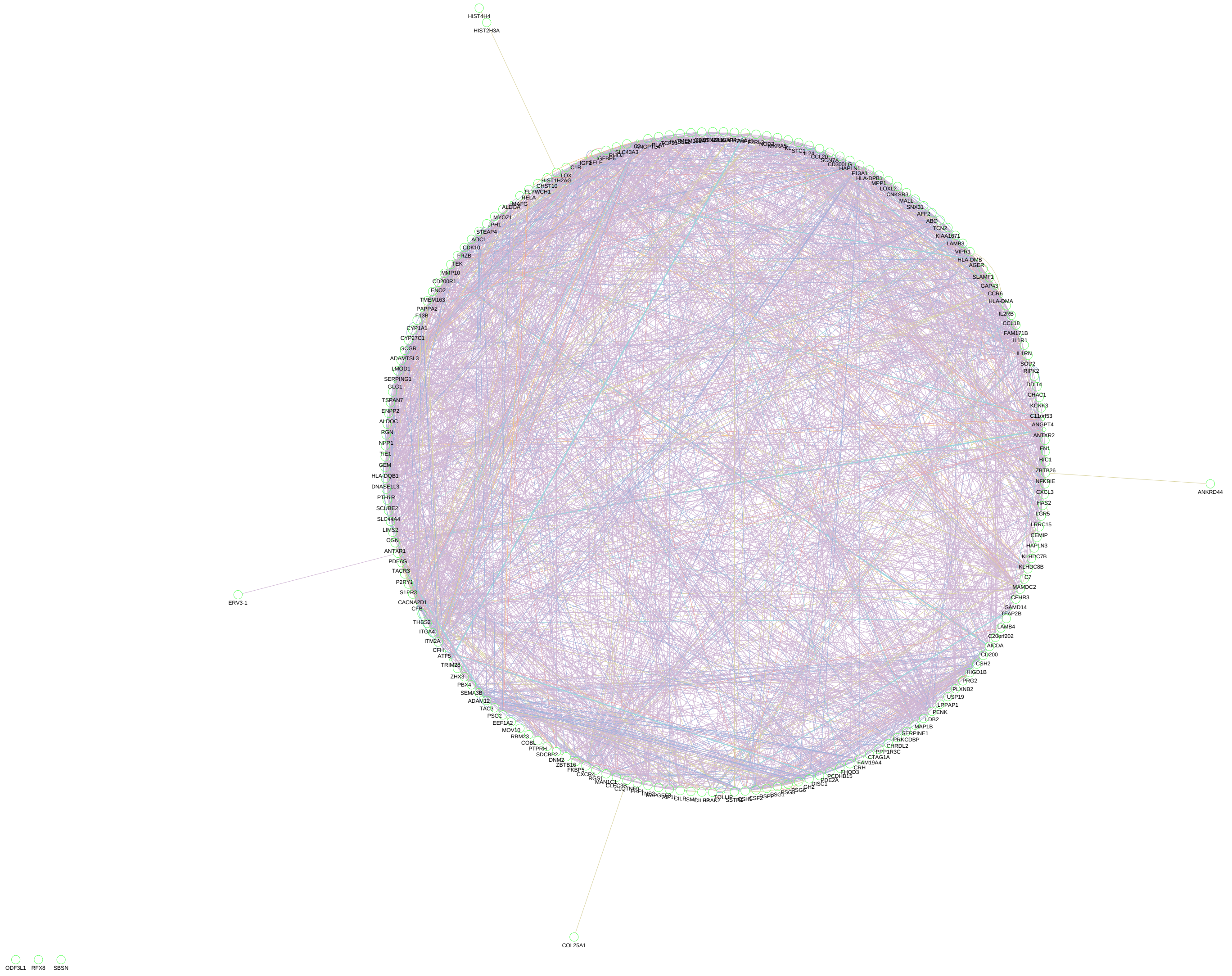

HIST4H4
HIST2H3A
SLC43A3
IGFBP6
IGF2
C1R
LOX
HIST1H2AG
CHST10
FLYWCH1
RELA
MAFG
ALDOA
MYOZ1
JPH1
STEAP4
AOC1
CDK10
FRZB
TEK
MMP10
CD200R1
ENO2
TMEM163
PAPPA2
F13B
CYP1A1
CYP27C1
GCGR
ADAMTSL3
LMOD1
SERPING1
GLG1
TSPAN7
ENPP2
ALDOC
RGN
NPP1
TIE1
GEM
HLA-DQB1
DNASE1L3
PTH1R
SCUBE2
SLC44A4
LIMS2
OGN
ANTXR1
PDE6G
TACR3
P2RY1
S1PR2
CACNA2D1
CFB
THBS2
ITGA4
ITM2A
CFH
ATF5
TRIM28
ZHX3
PBX4
SEMA3B
ADAM12
TAC3
PSG2
EEF1A2
MOV10
RBM23
COBL
PTPRH
SDCBP2
DNM2
ZBTB16
FKBP5
CXCR4
MAN1C1
CLEC3B
STC1
IL24
HAPLN1
HLA-DPB1
F13A1
MFP1
LOXL2
CNKSR3
MALL
SNX31
AFF2
ABO
TCN2
KIAA1671
LAMB3
VIPR1
HLA-DMB
AGER
SLAMF1
GAPDH
CCR6
HLA-DMA
IL2RB
CCL18
FAM171B
IL1R1
IL1RN
SOD2
RIPK2
DDIT4
CHAC1
KCNK3
C11orf53
ANGPT4
ANTXR2
FN1
HIC1
ZBTB26
NFKBIE
CXCL3
HAS2
LGR5
LRRC15
CEMIP
HAPLN3
KLHDC7B
KLHDC8B
C7
MAMDC2
CFHR3
SAMD14
TFAP2B
LAMB4
C20orf202
AICDA
CD200
CSH2
HIGD1B
PRG2
PLXNB2
USP19
LRPAP1
PENK
LDB2
MAP1B
SERPINE1
PRKCDBP
CHRDL2
PPP1R3C
CTAG1A
FAM19A4
CRH
PCDHB15
PDE2A
DISC1
ANKRD44
ERV3-1
COL25A1
ODF3L1
RFX8
SBSN

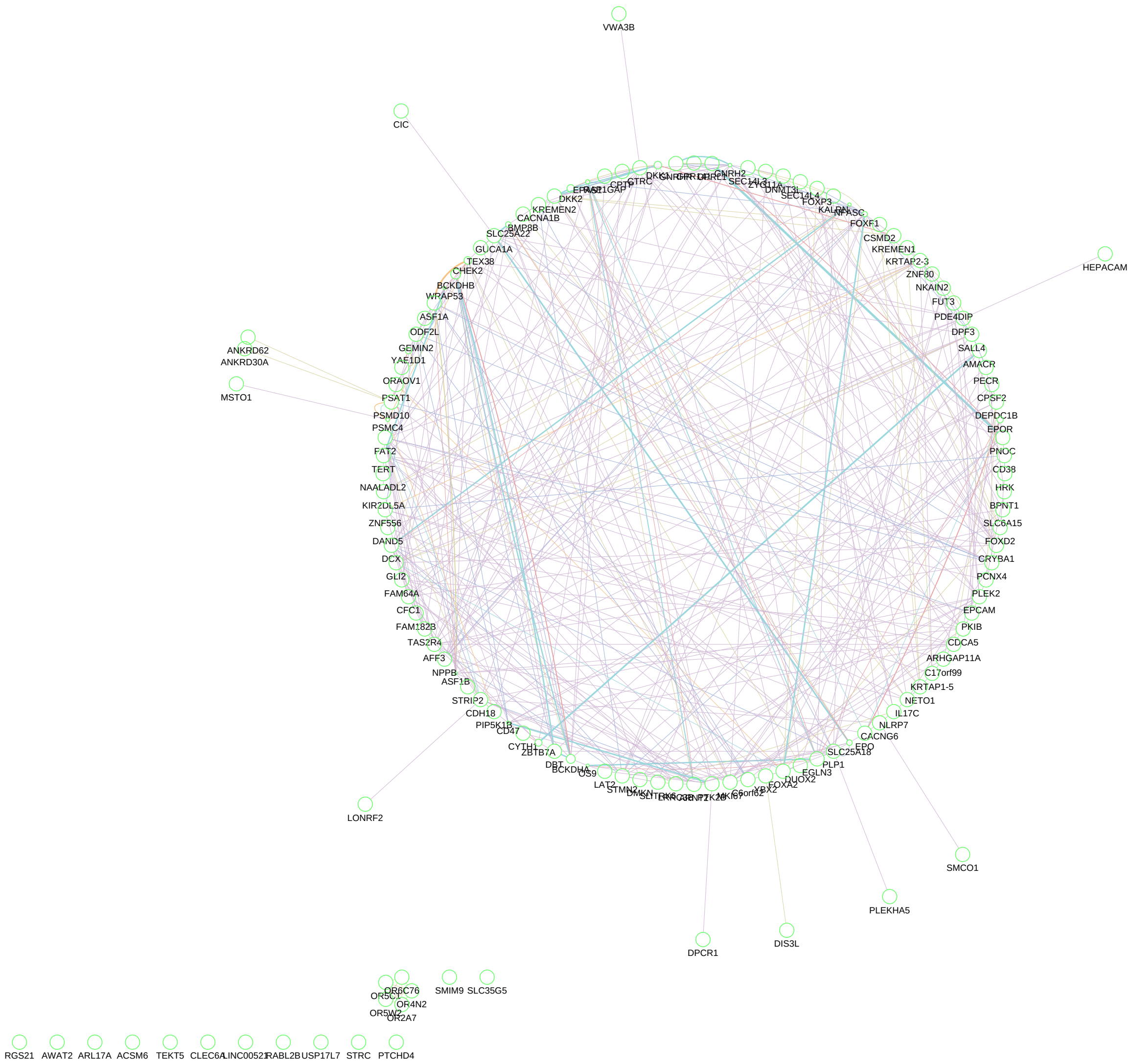
VWA3B
CIC
HEPACAM
ANKRD62
ANKRD30A
MSTO1
DKK1
CTRC
CPT1C
GNRH2
SEC14L3
DNMT3L
SEC14L4
FOXP3
KALRN
NFASC
FOXF1
CSMD2
KREMEN1
KRTAP2-3
ZNF80
NKAIN2
FUT3
PDE4DIP
DPF3
SALL4
AMACR
PECR
CPSF2
DEPDC1B
EPOR
PNOC
CD38
HRK
BPNT1
SLC6A15
FOXD2
CRYBA1
PCNX4
PLEK2
EPCAM
PKIB
CDCA5
ARHGAP11A
C17orf99
KRTAP1-5
NETO1
IL17C
NLRP7
CACNG6
EPO
SLC25A18
PLP1
EGLN3
DUOX2
FOXA2
YBX2
STMN2
LAT2
OS9
BCKDHA
DBT
ZBTB7A
CYTH1
CD47
PIP5K1B
CDH18
STRIP2
ASF1B
NPPB
AFF3
TAS2R4
FAM182B
CFC1
FAM64A
GLI2
DCX
DAND5
ZNF556
KIR2DL5A
NAALADL2
TERT
FAT2
PSMC4
PSMD10
PSAT1
ORAOV1
YAE1D1
GEMIN2
ODF2L
ASF1A
WRAP53
BCKDHB
CHEK2
TEX38
GUCA1A
SLC25A22
BMP8B
CACNA1B
KREMEN2
DKK2
LONRF2
SMCO1
PLEKHA5
DIS3L
DPCR1
SMIM9
SLC35G5
OR4N2
OR5W2
OR2A7
RGS21
AWAT2
ARL17A
ACSM6
TEKT5
USP17L7
RABL2B
STRC
PTCHD4

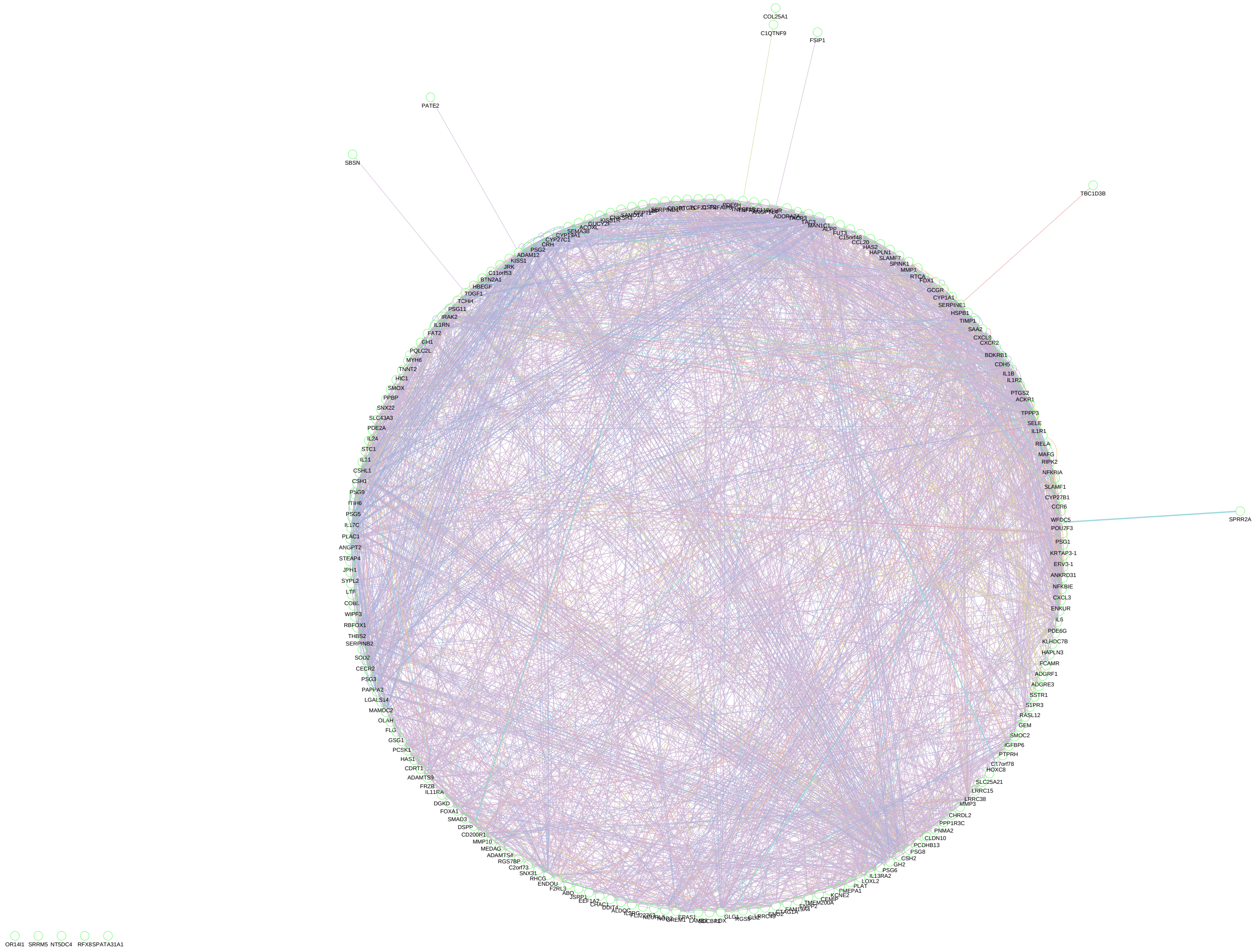

COL25A1
C1QTNF9
FSIP1
PATE2
SBSN
TBC1D3B
SPRR2A
OR14I1
SRRM5
NT5DC4
RFX8
SPATA31A1
ADORA2A
FUT3
C15orf48
CCL20
HAS2
HAPLN1
SLAMF7
SPINK1
MMP1
RTCA
FDX1
GCGR
CYP1A1
SERPINE1
HSPB1
TIMP1
SAA2
CXCL8
CXCR2
BDKRB1
CDH5
IL1B
IL1R2
PTGS2
ACKR1
TPPP3
SELE
IL1R1
RELA
MAFG
RIPK2
NFKBIA
SLAMF1
CYP27B1
CCR6
WFDC5
POU2F3
PSG1
KRTAP3-1
ERV3-1
ANKRD31
NFKBIE
CXCL3
ENKUR
IL6
PDE6G
KLHDC7B
HAPLN3
FCAMR
ADGRF1
ADGRE3
SSTR1
S1PR3
RASL12
GEM
SMOC2
IGFBP6
PTPRH
C17orf78
HOXC8
SLC25A21
LRRC15
LRRC38
MMP3
CHRDL2
PPP1R3C
PNMA2
CLDN10
PCDHB13
PSG8
CSH2
GH2
PSG6
IL13RA2
LOXL2
PLAT
PMEPA1
KCNE2
GLG1
ADAM12
KISS1
JRK
C11orf53
BTN2A1
HBEGF
TDGF1
TCHH
PSG11
IRAK2
IL1RN
FAT2
GH1
POLQ2L
MYH6
TNNT2
HIC1
SMOX
PPBP
SNX22
SLC43A3
PDE2A
IL24
STC1
IL11
CSHL1
CSH1
PSG9
ITIH6
PSG5
IL17C
PLAC1
ANGPT2
STEAP4
JPH1
SYPL2
LTF
COBL
WIPF3
RBFOX1
THBS2
SERPINB2
SOD2
CECR2
PSG3
PAPPA2
LGALS14
MAMDC2
OLAH
FLG
GSG1
PCSK1
HAS1
CDRT1
ADAMTS9
FRZB
IL11RA
DGKD
FOXA1
SMAD3
DSPP
CD200R1
MMP10
MEDAG
ADAMTS4
RGS7BP
C2orf73
SNX31
RHCG
ENDOU
F2RL3
ABO
PSG2
CRH
CYP27C1

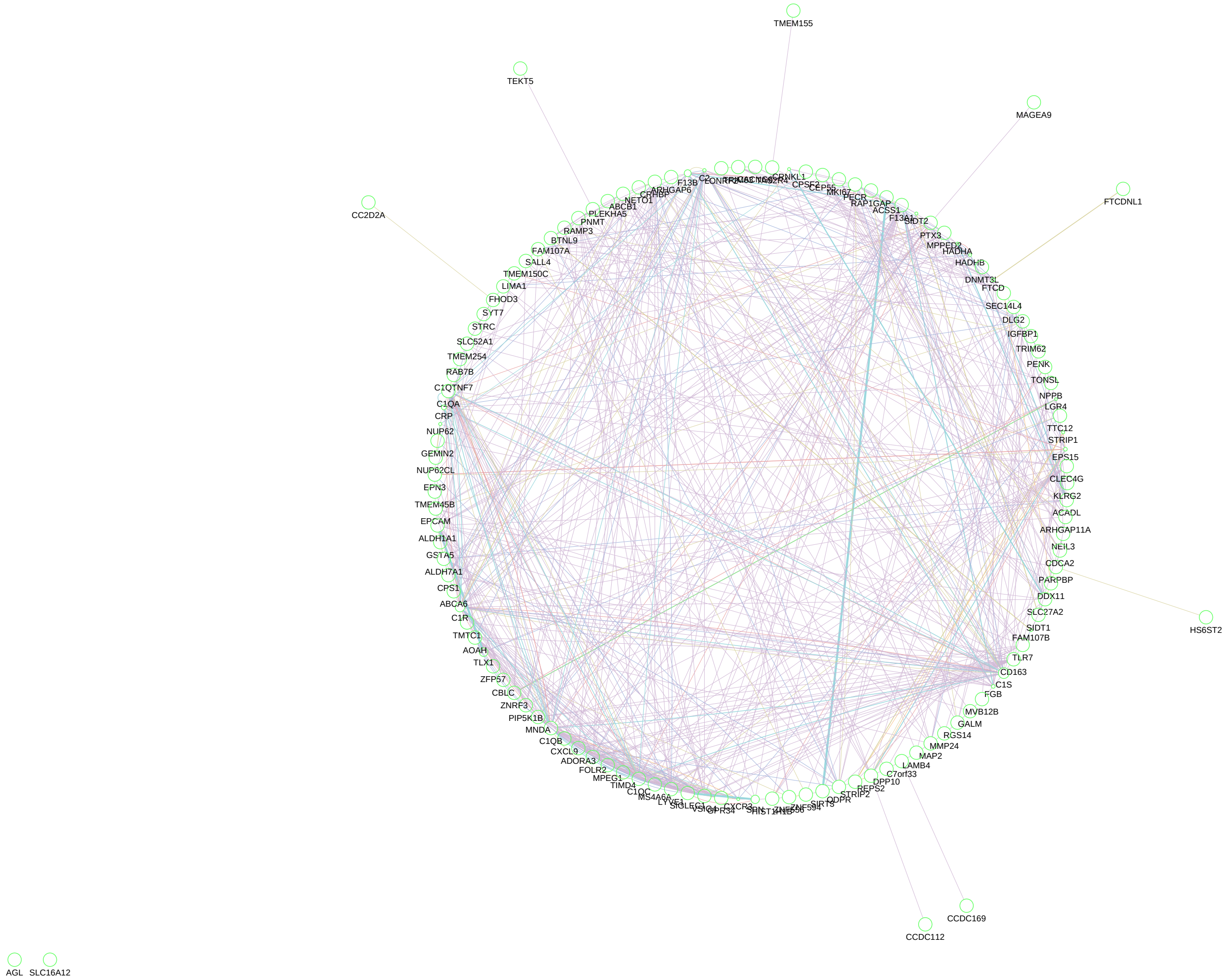
TMEM155
TEKT5
MAGEA9
FTCDNL1
CC2D2A
HS6ST2
CCDC169
CCDC112
AGL
SLC16A12
F13B
C2
CRNKL1
CPSF2
CEP55
MKI67
PECR
RAP1GAP
ACSS1
F13A1
SIDT2
PTX3
MPPED2
HADHA
HADHB
DNMT3L
FTCD
SEC14L4
DLG2
IGFBP1
TRIM62
PENK
TONSL
NPPB
LGR4
TTC12
STRIP1
EPS15
CLEC4G
KLRG2
ACADL
ARHGAP11A
NEIL3
CDCA2
PARPBP
DDX11
SLC27A2
SIDT1
FAM107B
TLR7
CD163
C1S
FGB
MVB12B
GALM
RGS14
MMP24
MAP2
LAMB4
C7orf33
DPP10
REPS2
STRIP2
ODPR
SIRT3
ZNF594
CXCR3
GPR34
SIGLEC1
MS4A6A
C1QC
TIMD4
MPEG1
FOLR2
ADORA3
CXCL9
C1QB
MNDA
PIP5K1B
ZNRF3
CBLC
ZFP57
TLX1
AOAH
TMTC1
C1R
ABCA6
CPS1
ALDH7A1
GSTA5
ALDH1A1
EPCAM
TMEM45B
EPN3
NUP62CL
GEMIN2
NUP62
CRP
C1QA
C1QTNF7
RAB7B
TMEM254
SLC52A1
STRC
SYT7
FHOD3
LIMA1
TMEM150C
SALL4
FAM107A
BTNL9
RAMP3
PNMT
PLEKHA5
ABCB1
NETO1
CRHBP
ARHGAP6
